# Two Thanksgivings

**Published:** 1875-11

**Authors:** 


### TWO THANKSGIVINGS.

Just one hundred years ago the nation was on the eve of an appointed Thanksgiving Day. Exactly what they had to be thankful for does not clearly appear. The summer of 1775 was a fearfully dry one, and vegetation was nearly destroyed. In the autumn, rain fell abundantly, and well-nigh universal sickness, accompanied with great mortality, followed. We were colonial dependents then, and were in the midst of a desolating war with Great Britain. Savage Indians were all around us, and our fate as a people hung suspended in so delicately poised a balance, that a breath might consign us to perpetual slavery. Threats were made by the attacking British forces to burn all the towns along the sea-coast, by means of their abundant navy. On the fourth day of November, Washington declared that the army was nearly destitute of cannon, and had only a small quantity of powder for the use of the musketry.

Yet they gave thanks, these sturdy ancestors of ours,—with as little to be thankful for, it may be, as ever mortals possessed. They had brave hearts, and that was about all. For this they thanked God; for this, and for their earnest belief in the justness of their cause. In this dark hour, deliverance was at hand. In half a year from that time they were a free and independent nation!

This Thanksgiving Day of one hundred years later, will dawn upon many hearts and homes rendered nearly joyless by sickness and poverty, where health and comparative wealth have abided. To such let the lesson come, that the history of nations and individuals shows that the daylight comes not less than the darkness. Courage, then! If we have not cause for thanksgiving in our present lot, let us find it in our brave hearts and in our hopes for a brighter day, as did our sires of a century ago.

### THE JOURNAL CHEAP.

Our readers should not forget that we receive subscriptions for all American and English magazines and papers at regular rates, and *give* HALL'S JOURNAL OF HEALTH to the amount of one quarter of the cash received. Thus, a person ordering any $4 publication, and remitting $4, will receive it one year, and the JOURNAL six months. By sending us $8, for other publications, they get the JOURNAL as a bonus for a whole year. This includes all magazines or papers published in America or England. Address all letters to "THE AMERICAN AND FOREIGN PUBLICATION COMPANY," 137 East Eighth Street, New York.

### COLD IN INFLAMMATIONS.

The Messrs. Hegeman, at their various drug-stores in this city, have for sale the peculiar Cone invented by Dr. Morris Mattson, the celebrated manufacturer of syringes for physicians' and domestic use. These Cones are so arranged that a current of cold water can be kept running through them for any length of time, with no effort on the part of the individual. No moisture comes in contact with the person, the only effect being that of dry cold, applied to the exact locality desired. Nothing more positively reduces the inflammation of piles than cold; and with the lessening of the inflammation, pain departs. The result from using this method of cure would be deemed magical, if it were not purely scientific. It does us good to recommend so valuable a device for the relief of one of the most annoying of maladies.

$77 A WEEK to Male and Female Agents in their locality. Costs NOTHING to try it, Particulars FREE. P. O. VICKERY & Co., Augusta, Maine.

